# Endothelial Microparticles in Uremia: Biomarkers and Potential Therapeutic Targets

**DOI:** 10.3390/toxins11050267

**Published:** 2019-05-13

**Authors:** Giane Favretto, Regiane Stafim da Cunha, Maria Aparecida Dalboni, Rodrigo Bueno de Oliveira, Fellype de Carvalho Barreto, Ziad A. Massy, Andréa Emilia Marques Stinghen

**Affiliations:** 1 Experimental Nephrology Laboratory, Basic Pathology Department, Universidade Federal do Paraná, Curitiba 81531-980, Brazil; gianefavretto@gmail.com (G.F.); regidacunha@gmail.com (R.S.d.C.); Fellype_Barreto@hotmail.com (F.d.C.B.); 2 Post-Graduation in Medicine Department, Universidade Nove de Julho, São Paulo 03155-000, SP, Brazil; dalboni@uni9.pro.br; 3 Division of Nephrology, Department of Internal Medicine, School of Medical Sciences Universidade de Campinas (UNICAMP), Campinas 13083-887, Brazil; rodrigobueno.hc@gmail.com; 4 Division of Nephrology, Ambroise Paré University Hospital, APHP, Boulogne-Billancourt, 92100 Paris, France; 5 France and Inserm U1018, Team 5, CESP, UVSQ, Paris-Saclay University, 94800 Villejuif, France

**Keywords:** Endothelial microparticles, cardiovascular disease, uremia

## Abstract

Endothelial microparticles (EMPs) are vesicles derived from cell membranes, which contain outsourced phosphatidylserine and express adhesion molecules, such as cadherin, intercellular cell adhesion molecule-1 (ICAM-1), E-selectin, and integrins. EMPs are expressed under physiological conditions and continue circulating in the plasma. However, in pathologic conditions their levels increase, and they assume a pro-inflammatory and pro-coagulant role via interactions with monocytes; these effects are related to the development of atherosclerosis. Chronic kidney dysfunction (CKD) characterizes this dysfunctional scenario through the accumulation of uremic solutes in the circulating plasma, whose toxicity is related to the development of cardiovascular diseases. Therefore, this review aims to discuss the formation of EMPs and their biological effects in the uremic environment. Data from previous research demonstrate that uremic toxins are closely associated with the activation of inflammatory biomarkers, cardiovascular dysfunction processes, and the release of EMPs. The impact of a decrease in circulating EMPs in clinical studies has not yet been evaluated. Thus, whether MPs are biochemical markers and/or therapeutic targets has yet to be established.

## 1. General Concept of Microparticles

Microparticles (MPs) were first described by Wolf in 1967 [1], when he observed a halo of debris surrounding activated platelets that he termed ‘platelet dust’. Since then, the available techniques for the detection of MPs have improved, and currently include flow cytometry, dynamic light scattering, nanoparticle tracking analysis, fluorescence correlation spectroscopy, immune blotting, mass spectrometry, transmission electron microscopy, and atomic force microscopy [2,3]. Unfortunately, the lack of uniformity with regards to the nomenclature of MPs has in some way hindered a better understanding of their role in pathophysiologic processes. Actually, different terms, such as nanoparticles, MPs, exosome-like vesicles, liposomes, and prostasomes, have been used depending on the sample source or the protocol used to isolate the MPs. Besides, MPs are often described as exosomes, smaller (40–100 nm) particles of endocytic origin, microparticles also known as microvesicles (100–1000 nm), from the reorganization of the plasma membrane, and apoptotic blebs (50–5000 nm) released by dying cells [2,4].

Cells release a variety of extracellular vesicles, including exosomes, MPs, and apoptotic bodies. MPs are found in the plasma and in other biological fluids of healthy individuals and their levels are altered in a pathological state. MPs present in the plasma are derived from several types of cells, such as endothelial cells, platelets, monocytes, neutrophils, and T-cells. They are vesicles derived from plasma membrane remodeling, virtually released by all cells in response to injury, apoptosis, or cellular activation. MPs are closely correlated with physiological processes. They may participate in the intercellular communication that helps in the maintenance of homeostasis under physiological conditions, or may initiate a deleterious process, e.g., an immune response, in the event of infection or in the presence of pathogens by transporting pathogenic constituents [5,6,7,8,9]. 

In general, MPs expose specific molecules to the parental cell. Depending on their origin, they may contain signaling molecules, including receptors, cytokines, mRNA, micro-RNA, and bioactive lipids [8,10]. The plasma membrane has a lateral organization of lipid raft domains that confers plasticity to the membrane. These lipid rafts are specialized regions of the cell membrane enriched with lipids and cholesterol, which allows them to be more rigid than the rest of the membrane. Lipid rafts also have the function of organizing the proteins into microdomains, transduction of signals, and transport via caveolin. This organization promotes the generation of unique responses resulting in the inclusion or exclusion of specific proteins and lipid species in cells, thereby explaining the different intravesicular compositions of MPs of the same cellular origin [6,11].

The release of MPs may induce cell signaling or may lead to the transfer of receptors between different cell types, since they carry parts of their cells of origin in their own membrane. This process occurs through the binding of the MPs to the membrane of the recipient cells; this binding may be of two types: (i) a ligand-receptor or (ii) a cell-adhesion interaction with subsequent MP internalization. These characteristics allow MPs to be able to mediate long-range signaling, which explains why they have been considered as emerging biomarkers for the diagnosis of various pathologies [5,10,12]. It has been demonstrated that circulating red cell MPs may be a potential blood biomarker for the differentiation between acute graft-versus-host disease and infection or sepsis after hematopoietic stem cell transplantation [13,14]. In recent years, the MPs from endothelial cells (EMPs) have been studied as endothelial injury markers, mainly in patients with cardiovascular disease, including the ones with chronic kidney disease (CKD) [15].

When exposed to a stimulus, endothelial cells can release MPs; endothelium-specific proteins, including endothelial cell-specific adhesion molecules, such as intercellular adhesion molecule (ICAM)-1, E-selectin, P-selectin, and platelet endothelial cell adhesion molecule, are found in these MPs [7,12]. Experimental data have shown that TNF-α stimulates human endothelial cells to increase the production of MPs expressing annexin-V, contain calcium and high levels of bone morphogenic protein-2, and are correlated with vascular calcification and osteogenic differentiation [16]. Burger et al. [17,18] showed that EMPs induce cell-to-cell signaling responses leading to inflammation, oxidative stress, and apoptosis [17,18]. High blood EMP concentrations found in some pathological conditions have been associated with inflammation and angiogenesis. EMPs may also contribute directly and indirectly to the blood coagulation cascade through the tissue factor (TF) [19,20,21].

## 2. Mechanisms of EMP Formation 

**Figure 1:**
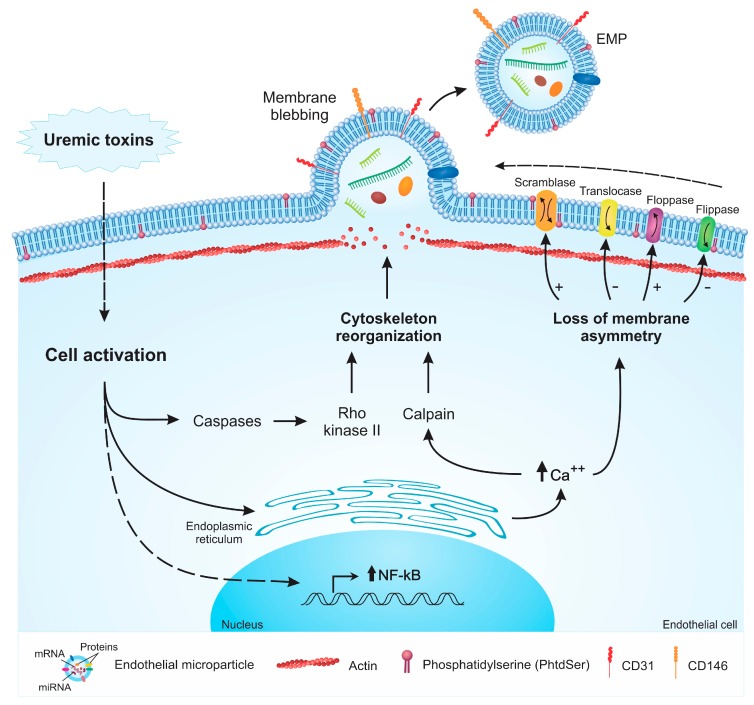
Schematic mechanisms of endothelial microparticle (EMP) formation induced by uremic toxins. Uremic toxins induce endothelial cell activation. This process activates the caspases, and consequently, rho kinase II, which leads to the reorganization of the cytoskeleton. Cell activation induces the release of calcium from the endoplasmic reticulum. The intracellular increase of calcium activates calpain, which in turn, induces the reorganization of the cytoskeleton. Calcium also leads to the activation or inhibition of proteins responsible for the maintenance of membrane asymmetry, causing the loss of this asymmetry. Cell activation elevates NF-κB expression. These processes cooperatively promote membrane blebbing and EMP formation.

Studies have shown that several compounds, such as TNF-α, glucose (high concentrations), thrombin, angiotensin II, uremic toxins (Figure 1), and others [22,23,24], are capable of inducing MP formation by endothelial cells. In addition, physical aspects such as shear stress can also lead to the release of MPs [25]. The lipid bilayer of the cell membrane is asymmetric due to the activity of transmembrane proteins such as flippases and floppases. The interior of the lipid bilayer is rich in phosphatidylethanolamine (PtdEtn) and phosphatidylserine (PhtdSer), while the outer side is rich in phosphatidylcholine and sphingomyelin. Cell activation from a stimulus or apoptosis leads to the loss of membrane asymmetry and cytoskeleton rearrangement, a process mediated by the increase in the intracellular levels of calcium released from the endoplasmic reticulum [7].

The increase in intracellular calcium deposition leads to the activation of receptors, which induces the release of calcium from the endoplasmic reticulum and the activation of caspases. The calcium flux activates calpain and phospholipase A2 [26]. Calcium is responsible for modulating the activity of two enzymes present in the plasma membrane, scramblase and aminophospholipid translocase, which results in the externalization of PhtdSer and activates cytosolic enzymes such as calpain [7,27,28]. The activation of caspases triggers Rho kinase II and MAP kinase signaling pathways, which contribute to cytoskeletal remodeling, enabling the formation of MPs [22,23]. 

Laminar shear stress also leads to the formation of EMPs. Atheroprone low shear stress conditions seem to increase the activity of Rho kinases and the extracellular signal-regulated protein kinases 1 and 2 (ERK1/2), resulting in increased MP generation, while the inhibition of these pathways reduces the formation of EMPs. In contrast, endothelial cells exposed to atheroprotective high shear stress conditions produce nitric oxide (NO), which downregulates the expression of ABCA1 flippase, a protein that modulates the membrane distribution of PhtdSer, and therefore, limits the formation of EMPs [25].

## 3. Characterization of EMPs

### 3.1. EMP Characterization by Flow Cytometry

**Figure 2:**
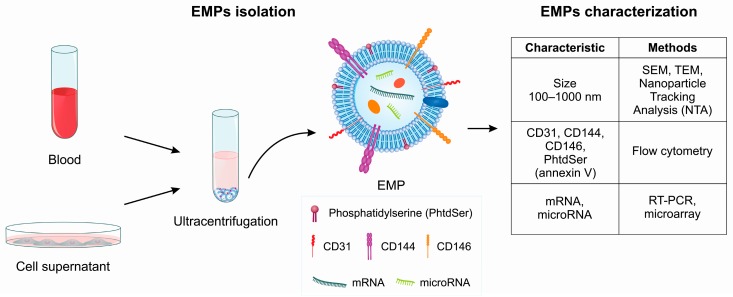
Schematic mechanism of the isolation and characterization of endothelial microparticles (EMPs). EMPs can be isolated from the blood or cell supernatants by ultracentrifugation. The EMPs can be characterized by their size (100–1000 nm), presence of PhtdSer in the membrane, and protein and nucleic acid compositions.

MPs can be isolated from blood circulation or cultured cells, such as endothelial cells (Figure 2). Flow cytometry is the most widely used method for characterizing MPs; however, some functional assays for MP analysis and characterization based on coagulation activation are also available [29]. Although flow cytometry provides useful information, some limitations of this method, such as low threshold for particle size detection, need for standardized instrument settings, and the requirement for appropriate antibodies against cell-associated antigens that are not expressed by other cell lineages, should be considered. For these reasons, the isolation of MPs from whole blood is a multi-step procedure, and many different process-dependent variables have been shown to affect the characterization and analysis of MPs. The outer leaflet of the MP membrane may express PhtdSer, which is a procoagulant phospholipid recognized by annexin-specific receptors [5]. The annexin binds to PhtdSer on the surface of the MPs [30]. Additionally, endothelial cells show a high expression of CD31^+^ and CD144^+^ [31]. Together, CD31^+^, CD144^+^ and annexin V^+^ expression have been described to enable the characterization of EMPs by flow cytometry.

Platelet-poor-plasma (PPP) obtained from citrated whole blood must be first centrifuged for 15 min at 500× *g* in order to obtain platelet-rich plasma (PRP); then this PRP must be centrifuged for 5 min at 14,000× *g* to pellet the platelets and obtain PPP. Then 50 μL from PPP must be incubated with 4 μL (the concentrations have been optimized by the titration of each reagent) of monoclonal antibodies against CD31^+^ and CD144^+^, followed by incubation with the annexin kit reagents, according to the manufacturer’s instructions, for 30 min in the dark at room temperature. After incubation, 450 μL of buffered saline solution (HBS; 20 mM HEPES, 150 mM NaCl, 2.5 mM calcium) must be added into the tube. The isotype antibodies must be used as negative controls. 

It is important to note that this method is common to characterize EMP. However, to obtain purified apoptotic bodies marked by Annexin V from EMP, it is necessary to use flow cytometric-based cell sorting. This methodology has the benefit of selecting individual particles of interest [32].

### 3.2. Sorting of EMP

Cell sorting allows the investigator to analyze quantitatively several fluorescence and light scattering parameters of individual particles and to purify those events with the desired characteristics for further study. No other technology can separate a heterogeneous cell suspension into purified fractions containing a single cell type with the speed and accuracy of high-speed cell sorters. For the sorting of EMPs, 250 µL PPP must be stained with CD31^+^, CD144^+^, and Annexin V^+^ and the corresponding isotype and negative controls. Stained plasma must be incubated for 45 min in the dark at room temperature according to the manufacturer’s suggestions. To sort EMPs, it is necessary to use flow cytometry cell sorting equipment. Vesicles between 100–1000 nm in diameter and stained with CD31^+^, CD144^+^, and Annexin V^+^ must be gated for sorting.

### 3.3. EMP Characterization by Electron Microscopy

Electron microscopy (EM) is currently used as a gold standard to characterize the morphology of microparticles, allowing the identification and measures of size of all different classes of extracellular microparticles. EM has a resolution around 0.5 nm, smaller than the size of the exosomes (40–100 nm), allowing a detailed visualization of the structural information of the MPs. EM provides semiquantitative information of the sample and it is not suitable for phenotyping. Another disadvantages are the changes in the characteristics of the MPs caused by vacuum procedures and standard dehydration procedures in EM, [3,10,33]. Variants of electron microscopy have been used to study the ultrastructure of MPs.

**Figure 3:**
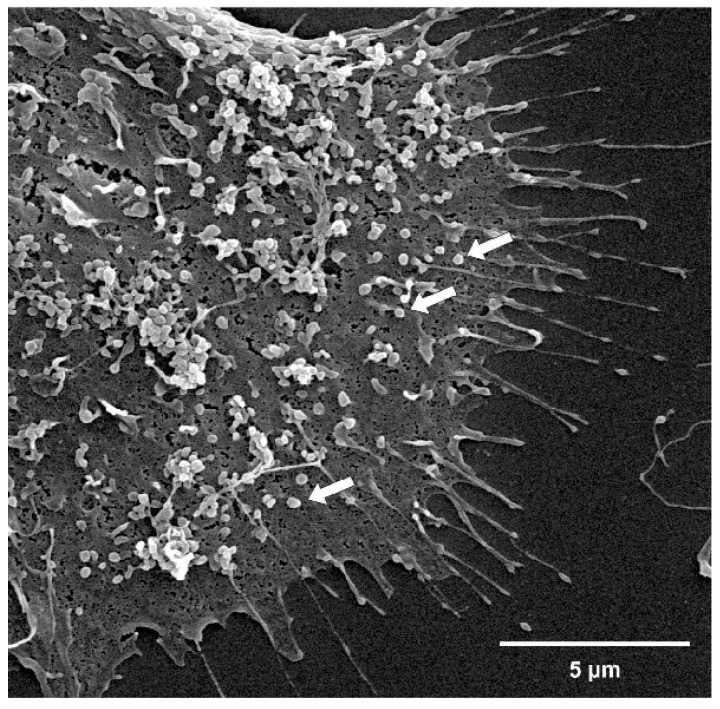
Scanning electron microscopy (SEM) showing endothelial cell microparticles on its surface. Arrows show microparticles. Magnitude: 15,000×.

Scanning electron microscopy (SEM) uses beams to scan the entire surface of the sample, generating topographic information. Sampling for SEM is simple when compared to other microscopy techniques. Samples should be fixed and followed by gradual dehydration with alcohol, followed by spraying a thin gold conductive layer to generate the images [33]. SEM imaging is carried out using 5 kV acceleration and a secondary electron (SE) detector. For SEM imaging, several randomly selected frames from each sample are captured for morphological observation and statistical purposes [34]. Figure 3 demonstrates EMP SEM.

Transmission electron microscopy (TEM) has the ability to characterize a single MP, providing information of biochemical and surface protein properties of the sample, and it is used to detect particles with 1 nm image resolution. It is the most widely used instrument to monitor the quality and purity of MPs-containing samples [35,36]. The disadvantages of TEM and SEM methods are that the preparation of samples is time-consuming, the MPs visualization can only be distinguished by their size and morphology, and by surface protein biochemical properties [37].

### 3.4. Nanoparticle Tracking Analysis (NTA)

Another commonly used method to directly detect MPs is NTA, a real-time method visualization that analyzes the nanoparticles in liquids in order to determine the size of MPs by light scattering using a light microscope. This method is used for sizing particles from 30 to 1000 nm. Brownian motion of the microparticles is individually screened and is dependent of the diffraction index of the submicron vesicles. Several videos are recorded, from which NTA software calculates, according to the Stokes–Einstein equation (Einstein relation), the size and total concentration of MPs present in the sample [3,38].

NTA has been gaining popularity in the MPs retraction analysis. Recently, Wang et al. [39] described novel methods to purify and detect MPs shed from endothelial cells and endothelial progenitor cells by combining microbeads with fluorescence quantum dots (Q-dots) coupled with nanoparticle tracking analysis (NTA). These novel methods provide ideal approaches for functional analysis and biomarker discovery [39]. Another study developed by Dragovic et al. [40] demonstrated that NTA is able to analyze total cellular MPs in human plasma using a fluorescent quantum dot-labeled cell tracker peptide [40]. Weber et al. [41] demonstrated that the characterization of individual MPs present in human whole blood showed a high level of reproducibility by fluorescence-based nanoparticle tracing analysis when compared to other methods and could be adjusted for characterization of MPs from cell culture supernatants [41].

## 4. Internalization and Signaling Pathways Induced by EMPs

EMPs interact with target cells (recipient) through membrane proteins and phospholipids [42]. This interaction allows the activation of membrane receptors and the internalization of the EMPs, which entails the transfer of active biomolecules and other contents into a recipient cell. In these cells, EMPs can lead to the activation of signaling pathways that result in changes of the cellular phenotype. In fact, studies have demonstrated that EMPs induce biological effects on recipient cells, such as endothelial cells, vascular smooth muscle cells (VSMCs), fibroblasts, monocytes, and others [11,43,44,45]. Among these biological effects, endothelial dysfunction induced by EMPs from endothelial cells exposed to indoxyl sulfate, a protein-bound uremic toxin, may be highlighted [43]. Although MPs have been considered an emerging topic in recent years, only a few studies have been devoted to EMPs [2,20].

**Figure 4:**
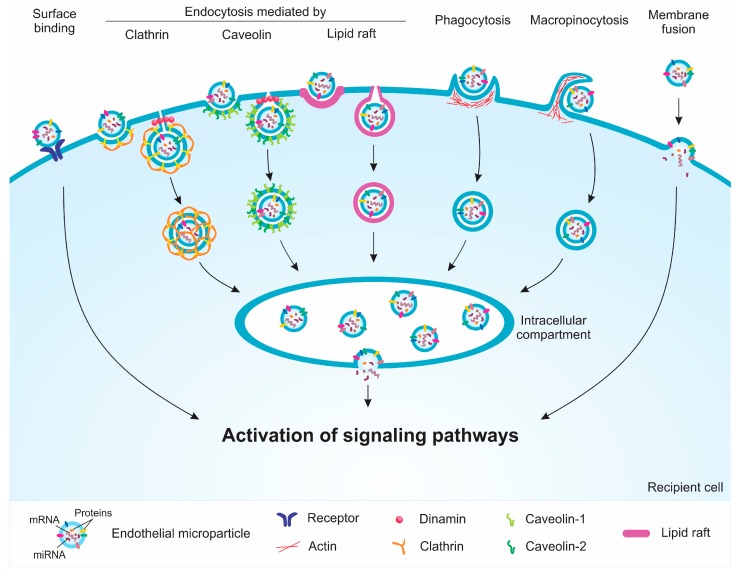
Schematic mechanisms of the internalization of endothelial microparticles (EMPs) by recipient cells. EMPs can interact with the surface of recipient cells through receptors, leading to the activation of signaling pathways. The uptake of the EMPs by the recipient cell can occur through clathrin-, caveolin-, and lipid raft-mediated endocytosis, phagocytosis, and macropinocytosis. These processes lead to the formation of vesicles that fuse with an intracellular compartment and induce the activation of various signaling pathways. The direct fusion between the membranes of the EMPs and the recipient cell may also occur.

MPs may possibly use more than one pathway to enter the recipient cells; however, studies have suggested that endocytic pathways are the main mechanisms of MP uptake. Thus, endocytosis inhibitors may be used to reverse or attenuate the biological effects induced by EMPs [46]. The internalization of MPs can occur by different mechanisms, such as caveolin-, clathrin-, or lipid raft-dependent endocytosis, phagocytosis, macropinocytosis, and direct membrane fusion (Figure 4). Each of these internalization mechanisms occur in a variety of different ways, particularly with regards to the proteins involved in each process. Caveolin-dependent endocytosis involves small invaginations in the membrane called caveolae; the protein caveolin-1 plays a crucial role in this process. It was observed that EMPs released by activated cells had an attenuated effect on caveolin-1 knockout endothelial cells or after treatment with dynasore, an inhibitor of caveola formation, compared to the case in wild-type endothelial cells [11]. Clathrin-dependent endocytosis is a process that involves the invagination of the membrane by accessory proteins, followed by the formation of clathrin-coated pits [46]. Similarly, lipid raft-dependent endocytosis involves membrane invaginations mediated by clathrin, caveolin, or other proteins in small regions rich in cholesterol, protein receptors, and sphingolipids [47]. Furthermore, phagocytosis is characterized by the formation of membrane extensions that surround the MPs to be internalized [48]. Macropinocytosis results from the rearrangement of the cytoskeleton, thus leading to the formation of membrane ruffles and allowing the internalization of extracellular materials such as MPs [49]. All these endocytic mechanisms result in the formation of a vesicle that merges with an intracellular compartment, in which membrane fusion and the release of the contents from MPs into the recipient cell could occur [50]. The mechanism by which this membrane fusion occurs is unclear, but an acid environment seems to contribute with this process [51]. Direct fusion of the MP membrane with the recipient cell is not so frequent; studies suggested that pH of the microenvironment may also contribute to this fusion [51,52,53].

Studies have identified several proteins that are important for the internalization of EMPs. However, the complete protein content of MPs remains difficult to be established. More than 300 proteins have been reported by proteomics, some of which are cytosolic and some membranous, and are dependent of the cell type [54]. The MP phenotypes vary according to cellular origin and parental cell response to stimulus [55,56]. Proteomic analysis of EMPs from starved endothelial cells demonstrated the presence of annexin I, an intracellular protein that is translocated to regions in the membrane that are rich in PhtdSer following apoptotic stimuli. Annexin I interacts with the PhtdSer receptor (PSR) expressed on endothelial cells, allowing the entry of EMPs into the target cell. The silencing of annexin I or PSR with small interference RNA significantly reduced the incorporation of EMPs by the recipient cells [30]. Another study demonstrated that the internalization of EMPs by endothelial cells involves proteins such as α_v_β_3_ integrin and lactadherin [42]. In addition, EMPs can bind to components of the extracellular matrix, including fibronectin, to which it binds through interaction with the α_v_ integrin. EMPs also activate matrix metalloproteinase-2, whose activity is important for vascular matrix remodeling [57].

The activation and incorporation of EMPs leads to a response by the recipient cells, such as inflammation and oxidative stress, leading ultimately to cellular dysfunction. EMPs released by TNF-α-treated endothelial cells induce the activation of the nuclear-factor kappa-b (NF-κB), a transcription factor involved in the expression of several pro-inflammatory molecules. An increased phosphorylation of the p65 fraction of NF-κB by lung endothelial cells after 30 min of exposure to EMPs from TNF-α-treated cells has been described. The expression of genes regulated by NF-κB, such as intercellular adhesion molecule-1 (ICAM-1), increased. An increase in the phosphorylation of epidermal growth factor receptor (EGFR), whose inhibition attenuated the activation of NF-κB, was observed [11]. EGFR also participates in the response of endothelial cells exposed to EMPs generated from angiotensin II-treated cells. In this case, both a higher production of reactive oxygen species (ROS) and expression of inflammatory molecules, such as vascular cell adhesion molecule 1 (VCAM-1) and CD31, were observed [58]. Moreover, EMPs produced after treatment with high glucose concentrations promoted the expression of von Wille-brand factor on the surface of endothelial cells; this contributed to a greater interaction between platelets and the endothelium [42]. Finally, a greater level of phosphorylation of ERK1/2 and Src has been reported in endothelial cells exposed to EMPs, suggesting that the activation of these pathways may also play a part in the response of cells to EMPs [18].

The type of stimulus that leads to EMP production seems to be important for the activation of signaling pathways in the recipient cell. In fact, in vitro analyses have demonstrated that EMPs could attenuate the endothelial inflammation induced by TNF-α. In this case, EMPs derived from starved cells transfer into a recipient cell, a functional microRNA-222 that can reduce the ICAM-1 expression. Interestingly, EMPs derived from cells treated with high concentrations of glucose contain lower amounts of microRNA-222, and therefore do not affect the ICAM-1 expression [59]. 

The role of EMPs on endothelial dysfunction and vascular inflammation has been studied in vivo. For this purpose, EMPs released from cells exposed to high glucose concentrations were administrated to apolipoprotein-E knockout mice in order to simulate diabetes. It was demonstrated that ICAM-1 and VCAM-1 expression was induced, resulting in increased macrophage infiltration in the vessel walls, which is a well-recognized factor linked to the pathogenesis of atherosclerosis. In agreement with these findings, in vitro studies demonstrated increased ROS production and the increased phosphorylation of p38, a signaling protein involved in endothelial activation [60]. Other studies have also shown that EMPs induce ROS production in endothelial cells [18,60,61,62]. This increase is explained, at least in part, by the activation of the NADPH oxidase pathway [18]. Moreover, the production and the bioavailability of NO is reduced, which has an effect on endothelium-dependent vasorelaxation [18,61]. Otherwise, it was recently described that EMPs from TNF-α-treated cells may have a protective effect against palmitate-induced oxidative stress in endothelial cells [63]. These findings suggest that EMPs could have an ambivalent role depending on the microenvironment.

EMPs can also modulate immune response processes such as monocytic activation. It has been demonstrated that EMPs, released from quiescent cells, have the miR-10a transferred to the monocytes, which results in the inhibition of the NF-κB pathway in this cell type [64]. This shows that the bioactive molecule composition of the EMPs is important for the activation or suppression of signaling pathways. In vitro studies have also shown that EMPs derived from TNF- α-treated endothelial cells are capable of increasing the production of the tissue factor-dependent procoagulant activity in monocytes [45]. EMPs may also influence the activation of plasmacytoid dendritic cells, by increasing the expression of the inflammatory cytokines interleukins-6 (IL-6) and IL-8 [65]. Moreover, Wheway et al. [66] demonstrated that EMPs interact by releasing their content into CD4^+^ and CD8^+^ cells, increasing cell proliferation. This study also showed that EMPs express important molecules for the activation of T cells, such as CD40, ICOSL, and MHC II, which suggests that EMPs could modulate the cellular response [66].

## 5. Uremia and EMPs

In CKD settings, it has been shown that the generation of MPs may not only be a consequence of this disorder, but also be a major cause of the onset of pathological processes [16,67]. The constant injury of the endothelium promoted by inflammation, uremic toxins, and other mechanisms induce endothelial dysfunction, being responsible for the production of MPs and consequent vascular calcification [68,69]. In fact, several studies have demonstrated that uremic toxins such as phosphate, *p*-cresol, *p*-cresyl sulfate, indoxyl sulfate, and homocysteine are closely correlated with the induction of MPs [69,70,71,72,73,74].

Calcium and phosphate demonstrated to be present in uremia-related endothelial dysfunction in CKD patients, and are also directly associated with high circulating levels of EMPs [69]. Hyperphosphatemia caused by the intracellular accumulation of phosphate in patients with CKD is considered a crucial factor for the development of cardiovascular disease; it may lead to a greater increase of circulating MP levels [72,73]. Intracellular accumulation of phosphate mediated by PiT1/slc20a1 transporters resulted in increased membrane blebbing and an increase in the release of MPs. Pi-induced MPs have procoagulant properties and are involved in vascular and thrombotic events [72].

*P*-cresyl sulfate increases endothelial permeability and is closely correlated with the Rho kinase protein, which is responsible for the reorganization of the cytoskeleton and consequent MP formation [27,74,75]. *p*-Cresol and *p*-cresyl sulfate are associated with endothelial dysfunction and the release of MPs, with serum *p*-cresol levels being independent of the number of circulating MPs in patients with CKD and patients undergoing hemodialysis. In contrast, *p*-cresyl sulfate (0.1, 0.5, and 1 mmol/L) induces dose-dependent MP formation in vitro [69]. Endothelial cells treated with p-cresol (40 μg/mL) and indoxyl sulfate (256 μg/mL) in the free fraction and protein-bound in the presence of human serum albumin (HAS) have an important impact on endothelial activation and the in the release of EMPs [68].

Indoxyl sulfate is capable of activating the endothelium, thus inducing MPs in the circulation, it also alters the endothelial repair process in patients with CKD, and pathologies associated with endothelial dysfunction, such as CKD and antiphospholipid syndrome. Indoxyl sulfate-induced (256 μg/mL) MPs have miRNA and other endothelium-characteristic molecules that participate in signaling pathways involved in oxidative stress and cellular apoptosis, hindering endothelial regeneration [43]. Indoxyl sulfate-induced EMPs influence the neointimal hyperplasia and smooth muscle cell proliferation through phosphorylation of TGF-β downstream molecules in VSMCs. Therefore IS-induced (250 μg/mL) EMPs have a critical role in not only stimulating the TGF-β signaling pathway in VSMCs, but also in neointimal formation [76].

EMPs released by cells exposed to indoxyl sulfate (250 μg/mL) promoted functional loss in endothelial progenitor cells, which are bone marrow-derived cells with angiogenic properties that are important for endothelial repair. These EMPs can modulate the classic endothelial roles of progenitor cells as colony forming units and form new vessels, as well as increase the expression of NF-κB and p53 by the endothelial progenitor cells in vitro. Indoxyl sulfate is capable of inducing endothelial vesiculation with different membrane characteristics, miRNAs and other molecules, which makes maintaining of vascular homeostasis of endothelial progenitor cells not fully functional [43]. Clinical studies have also associated the increase of EMPs with the reduction of endothelial progenitor cells in patients with CKD [44,77]. 

High levels of EMPs in chronic uremic renal patients (CRI) in non-dialyzed or hemodialyzed (HD) patients. Elevation of EMPs can be compared to other vascular pathologies mediated by uremia and consequent endothelial dysfunction [68]. EMPs seem to participate in the disruption of vascular homeostasis promoted by uremic toxicity, which could contribute to uremia-related cardiovascular disease. Actually, EMPs isolated from CKD patients induced the expression of osteocalcin, an osteogenic protein linked to vascular calcification, in endothelial progenitor cells, VSMCs, and fibroblasts [44].

## 6. Microparticles and Cardiovascular Disease

Endothelial cells play an important role in the development of cardiovascular diseases in response to activation and release of MPs. Several studies have demonstrated the association between high circulating levels of MPs and cardiovascular events inflammation-related. MPs are involved in thrombosis, angiogenesis, autophagy, cell survival, and apoptosis, which are important events in homeostasis and in progression of cardiovascular diseases [12,78]. Indeed, it was demonstrated that MPs are capable of mediating long-range signaling, acting on different targets from those of their own cellular origin. Depending on their cellular origin and signaling, MPs may exert different stimuli on vascular endothelial cell [67,73,78]. As a matter of fact, Faure et al. [68] demonstrated that patients who do not have a history of cardiovascular disease have relatively similar levels of EMPs compared to patients with a history of cardiovascular disease. The study did not exclude the possibility that high levels of EMPs in patients with uremia is a result of vascular diseases, since it is already well established that patients with CKD have an accelerated progression of atherosclerosis related to endothelial dysfunction [68].

Patients with age-related cardiovascular diseases such as congestive heart failure, coronary artery disease, peripheral vascular disease, and cerebral ischemia have an increased number of circulating MPs [21]. In atherothrombotic cardiovascular diseases, increased levels of MPs derived from platelets, endothelial cells, monocytes, granulocytes, and red blood cells may be detected [27].

MPs originating from monocytes, platelets, and lymphocytes induce endothelial dysfunction by reducing NO levels and increasing oxidative stress, activate proinflammatory cytokines such as IL-1, IL-6, and IL-8, and may interact with adhesion molecules such as E-selectin, ICAM-1, and VCAM-1 [79]. EMPs selectively affect the signal transduction pathway and the release of NO by endothelial cells, which leads to the generation of cyclical guanosine monophosphate (cGMP) in VSMCs; such cellular processes are important for endothelium-dependent vascular relaxation. Circulating EMPs may act as specific inhibitors of the synthesis of NO by endothelial cells, through negatively affecting the acetylcholine-induced release of cGMP, an important compound for the biosynthesis of NO [67]. 

Elevated levels of circulating MPs have been detected in cardiovascular and immune-mediated diseases. MPs of patients with myocardial infarction induce endothelial dysfunction through the impairment of the NO pathway in endothelial cells, but without altering the expression of endothelial NO synthase (eNOS). However, this effect seems to depend on the cellular source of MPs. T cell-derived MPs may induce endothelial dysfunction by altering the expression of the eNOS and caveolin-1 genes. In addition, MPs may promote the expression of proinflammatory proteins involved in changes in vascular contractility [80].

## 7. Therapeutic Interventions and Modulation of the Levels of MPs

Clinical and experimental evidence suggests the use of MPs as biochemical markers or therapeutic targets [81]. Although the amount of consistent data from clinical trials is relatively small, interesting studies have suggested the use of polyphenolic compounds, simvastatin, kidney transplantation, immunosuppressant drugs, and convective hemodialysis as potential strategies for MP modulation [82,83].

Ammollo et al. [82] investigated the influence of polyphenolic compounds (flavonoids, phenolic acid, resveratrol) on MPs from whole blood. For 3 weeks, grapes (5 g/kg/day) or placebo were offered to 20 and 10 healthy volunteers, respectively. After 3 weeks, grape consumption was associated with a decrease of thrombin generation, decreasing the number and activity of procoagulant MPs. The antithrombotic effect was sustained after 4 weeks of washout time. Based on these findings, it was suggested that the grape compounds sustained anticoagulant and profibrinolytic effects through the modulation of procoagulant MPs [82].

Almquist et al. [84] tested the effects of simvastatin alone or with ezetimibe on MPs from platelets, monocytes, and endothelial cells for 8–10 weeks, in a cohort of 39 patients with diabetes mellitus with or without CKD. In 18 patients who had diabetes mellitus and CKD stages 3–4 the levels of almost all types of MPs tested were elevated, when compared to diabetic patients without CKD. Administration of simvastatin (40 mg daily) was associated with the reduction of the procoagulant effects of MPs in the diabetic CKD patients, while the combination of simvastatin and ezetimibe had no further effect on the levels of MPs. These results suggest that simvastatin might have beneficial action on hypercoagulability in this high-risk population by modulating the MP concentration [84].

Kidney transplantation and immunosuppressive therapy also seem to play a role in regulating the levels of MPs. Al-Massarani et al. [85] studied the effects of two different immunosuppressive regimens on endothelial biomarkers, including EMPs, in 52 patients who underwent kidney transplantation, in reference to 50 healthy subjects as the controls. They found a favorable impact of kidney transplantation after 12 months on endothelial markers, expressed among other factors, through the reduction of the levels of EMPs, which reached normal values. Of note, cyclosporine microemulsion/azathioprine seems to have more pronounced positive effects on the concentration of EMPs, when compared to tacrolimus/mycophenolate mofetil [85]. 

Ramirez et al. [86] investigated whether on-line hemofiltration would be an efficient strategy to remove uremic toxins, including EMPs in comparison to high-flux hemodialysis. In this study, 15 stable patients on high-flux hemodialysis were switched to on-line hemofiltration for 4 months and thereafter switched back to high-flux hemodialysis. A decrease in the numbers of endothelial MPs and endothelial progenitor cells was observed during the on-line hemofiltration treatment period, which signals the attenuation of endothelial damage. Interestingly, after returning to the previous therapy, their levels increased their basal values. These observations are consistent with those of a prospective crossover study by Ariza et al. [83], which demonstrated lower levels of apoptotic EMP, when comparing post-dilution high convective transport techniques and high-flux hemodialysis.

## 8. Conclusions

MP formation has been widely studied in several physiological and pathological conditions such as coagulation disturbances, diabetes, hypertension, cardiovascular diseases, uremia, and others. Their formation is complex and involves an increase in the intracellular calcium deposition, which in turn increases the release of MPs from several types of cells, including endothelial cells. Endocytic pathways are the main mechanisms of MP uptake by a variety of other recipient cells; it is possible that MPs use more than one pathway to enter the recipient cells. In the intracellular space, MPs activate several inflammatory and oxidative stress pathways, ultimately leading to cellular dysfunction. In uremic patients, a constant attack on the endothelium by uremic toxins, such as the protein-bound p-cresyl sulfate and indoxyl sulfate and inorganic phosphate, induces the release of EMPs from endothelial cells; however, the details of this mechanism have not been fully elucidated. Current evidence, though limited, has shed some light on the possible strategies to reduce EMP release, which seems to share the common pathway of ameliorating the uremia-related pro-inflammatory state. It is noteworthy that the impact of reducing the levels of EMPs on clinical outcomes has not yet been evaluated. Whether MPs are biochemical markers or therapeutic targets remains to be established.
